# Evaluation of the Impact of Alveolar Nitrogen Excretion on Indices Derived from Multiple Breath Nitrogen Washout

**DOI:** 10.1371/journal.pone.0073335

**Published:** 2013-09-09

**Authors:** Niklas Nielsen, Jorgen G. Nielsen, Alex R. Horsley

**Affiliations:** 1 Institute of Mathematics and Computer Science, University of Southern Denmark, Odense, Denmark; 2 Innovision Ltd., Odense, Denmark; 3 Institute of Inflammation and Repair, University of Manchester, Manchester, United Kingdom; 4 Manchester Adult Cystic Fibrosis Centre, University Hospital of South Manchester, Manchester, United Kingdom; University of Pittsburgh, United States of America

## Abstract

**Background:**

A large body of evidence has now accumulated describing the advantages of multiple breath washout tests over conventional spirometry in cystic fibrosis (CF). Although the majority of studies have used exogenous sulphur hexafluoride (SF_6_) as the tracer gas this has also led to an increased interest in nitrogen washout tests, despite the differences between these methods. The impact of body nitrogen excreted across the alveoli has previously been ignored.

**Methods:**

A two-compartment lung model was developed that included ventilation heterogeneity and dead space (DS) effects, but also incorporated experimental data on nitrogen excretion. The model was used to assess the impact of nitrogen excretion on washout progress and accuracy of functional residual capacity (FRC) and lung clearance index (LCI) measurements.

**Results:**

Excreted nitrogen had a small effect on accuracy of FRC (1.8%) in the healthy adult model. The error in LCI calculated with true FRC was greater (6.3%), and excreted nitrogen contributed 21% of the total nitrogen concentration at the end of the washout. Increasing DS and ventilation heterogeneity both caused further increase in measurement error. LCI was increased by 6–13% in a CF child model, and excreted nitrogen increased the end of washout nitrogen concentration by 24–49%.

**Conclusions:**

Excreted nitrogen appears to have complex but clinically significant effects on washout progress, particularly in the presence of abnormal gas mixing. This may explain much of the previously described differences in washout outcomes between SF_6_ and nitrogen.

## Introduction

Over the last ten years a growing body of evidence has demonstrated the value of lung physiology measurements derived from multiple breath washout tests (MBW), in particular the lung clearance index (LCI) [1], [2]. LCI has been shown to be reproducible, repeatable, more sensitive to early airways disease and more sensitive to interventions in patients with cystic fibrosis (CF) than spirometry [2], [3], [4], [5], [6]. There are additional advantages in children that have been well described, which relate to the ease of performing the test and the stable normal range [7]. LCI is now moving from a research tool performed in selected laboratories, on equipment prepared in-house, to a mainstream clinical measurement and there are currently at least three commercially available devices specifically designed to measure LCI, alongside numerous others that offer this facility as an option.

Measurement of LCI is in principle straightforward, and depends upon the washout of an inert tracer gas from the lungs, with no interference from gas exchange. The choice of the inert gas however is an important one, with implications both for the performance and the interpretation of the test. The recent consensus statement on MBW described three different tracer gases that have been used to measure LCI [8]: helium, sulphur hexafluoride (SF_6_) and nitrogen (N_2_). Differences in molar mass affect the diffusion front, which lies more distally for SF_6_ than for the much lighter helium. This difference has potential to be exploited to explore the site of lung pathology [9], though in practice the majority of studies on MBW so far have involved either SF_6_ or nitrogen as the tracer gas. A practical advantage with use of nitrogen washout is that whereas SF_6_ must first be washed into the lungs, nitrogen is already resident. This therefore requires that multiple breath nitrogen washout (MBNW) is performed with a washout gas free of nitrogen (typically 100% oxygen is used), whilst SF_6_ washout is performed with room air. A more fundamental difference exists between nitrogen and SF_6_ (and He) MBW however: nitrogen is not truly inert. There is significant solubility in blood and tissue [10] and the body contains large volumes of dissolved nitrogen [11]. The majority of this is stored in fat tissue [12], with a long washout time constant that is unlikely to interfere with accuracy of LCI testing, since this typically takes no more than 5 minutes to complete. Significant amounts of nitrogen however are also stored in the blood and well perfused tissues [11], [12], [13], and this contribution to expired nitrogen has so far been ignored. The consensus statement [8] referred to data from two papers to explain why allowance for body nitrogen was not required [12], [14].

More recently, data have been published, which seem to show a large and highly significant separation between both LCI and functional residual capacity (FRC) measurements performed using the different tracer gases [15]. Washout was performed simultaneously on both helium and SF_6_, and the difference was between both of these and subsequent nitrogen washout, suggesting a difference between lung volumes measured by nitrogen and those measured by exogenous gases in general. The authors concluded that this difference related to the washout of resident tracer gas from poorly ventilated lung units that were not detected well by exogenous tracer. Alternative hypotheses are that this difference could be related to the technology involved (since washouts were performed using very different apparatus), to the effects of body nitrogen or to the use of pure oxygen during washout. Although the earlier studies did not show a major impact of allowing for the effect of body nitrogen in healthy lungs [14], the process may be quite different in those with ventilation heterogeneity. During the later phases of the washout the best ventilated lung regions will have been washed clear of tracer and in the nitrogen washout will contain 100% O_2_. Nitrogen will move out of solution down a concentration gradient, and will thus be preferentially excreted from the best ventilated lung regions. This difference between SF_6_ and nitrogen measurements may therefore come paradoxically from the best ventilated lung regions rather than being alveolar nitrogen washed slowly from the most severely affected lung regions.

It is very hard to address the question of the influence of nitrogen excretion from the body directly using both tracers simultaneously, since measurement of nitrogen by mass spectrometry in the presence of high levels of oxygen requires different settings to those used to measure SF_6_. We hypothesised that data on nitrogen excretion during oxygen breathing [12], [13] could be used to inform a two compartment lung model, which could then be used to explore the effects of ventilation heterogeneity on nitrogen excretion.

The aims of this study were thus:

1. To generate a two compartment lung model containing variable components representing both dead space (DS) and ventilation heterogeneity that generates physiologically realistic LCI values.
2. To apply clinical data on nitrogen excretion to this lung model to mimic the *in vivo* effects of nitrogen excretion on the MBNW curve and outcomes (FRC and LCI), and to assess how increases in DS and ventilation heterogeneity affect this.
3. To estimate the potential impact of nitrogen excretion on accuracy of washout outcomes (FRC and LCI) and washout progression in children.

## Methods

### Lung Model

**Figure 1:**
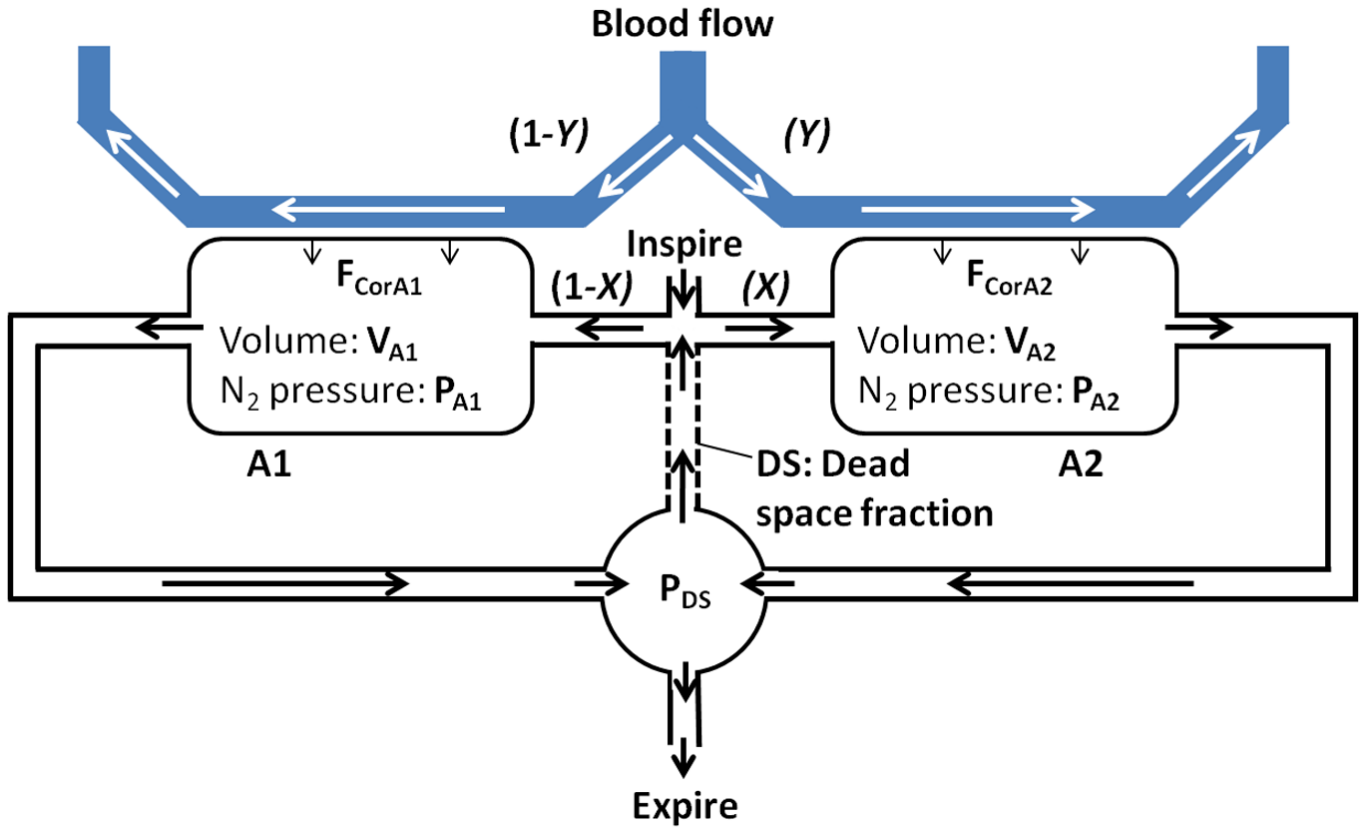
Two compartment lung model incorporating nitrogen excretion. There are two alveolar compartments, A1 and A2, with volumes V_A1_ and V_A2_ respectively. The proportion of the ventilation and blood flow received by A2 are defined as X and Y respectively. A proportion of expiration representing dead space is re-circulated (DS). P_A1,_ P_A2_ and P_DS_ are the nitrogen partial pressures in the two alveolar and DS compartments respectively and F_CorA1_ and F_CorA2_ the rates of nitrogen excretion from the blood into the two alveolar compartments.

The lung model used for the simulation consisted of two parallel compartments with a common serial dead space (see Fig. 1). Ventilation was simulated as continuous flow through each of the two compartments. Different fractional ventilation of the two compartments allowed assessment of the effect of ventilation heterogeneity on nitrogen excretion. The influence of DS was simulated as recirculation of a fraction corresponding to the assumed DS ventilation relative to total ventilation. This allowed control over the two major parameters that determine LCI: the degree of specific ventilation heterogeneity and the degree of airway dead space.

Mass balances for nitrogen in compartments A1, A2 and DS were determined by:
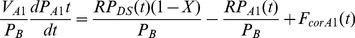
(1)

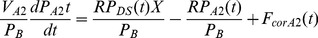
(2)

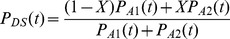
(3)Where:

*V_A_*_1_ and *V_A_*_2_ are volumes of the two parallel compartments A1 and A2 respectively. Added together, these make up the functional residual capacity (FRC) of the model.

*P_B_* is the barometric pressure.

*P_A_*_1_(*t*) and *P_A_*_2_(*t*) are the nitrogen partial pressures in the two compartments A1 and A2.

*R* is ventilation rate.

*X* is the fraction of ventilation going to compartment A2.

*F_CorA_*_1_(*t*) and *F_CorA_*_2_(*t*) are the rates of nitrogen excretion from the blood into each of the two compartments.

*P_DS_*(*t*) is the nitrogen partial pressure in the dead space compartment, and hence also the “expired” nitrogen signal.

In multiple breath washouts, nitrogen pressure starts at atmospheric pressure. This is the same nitrogen partial pressure as the initial pressure in blood and tissue, and thus initially nitrogen backflow across the alveolar membrane will be zero. We have corrected the clinical data in the following way:
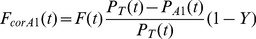
(4)

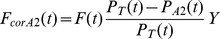
(5)Where:

*F*(*t*) is the total nitrogen excretion from tissue and blood into a lung with zero alveolar nitrogen partial pressure.

*P_T_* (*t*) is the tissue and blood partial pressure of nitrogen.

*Y* is the fraction of blood flow going to compartment *A*2.

Since nitrogen is only dissolved in tissue and blood (not chemically bound) we assume that its’ partial pressure decreases linearly with the cumulative excretion of nitrogen:
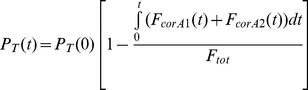
(6)Where *F_tot_* is the total amount of nitrogen in the blood and tissue at the start of nitrogen washout. This was set at 1000 ml [13].

The flow of nitrogen from the blood and tissues across the alveolar membrane, *F’(t)*, was based upon data provided by Lundin [12] obtained in experiments where nitrogen pressure in the alveoli was kept at zero. This involves the combination of two exponential equations, describing elimination of nitrogen from: (i) blood and tissues, and (ii) from muscles. A third exponential, describing elimination from fat, was omitted from this model as the time course was too long to have significant impact on nitrogen excretion during an LCI assessment. The equation describing nitrogen elimination is thus:
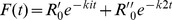
(7)Where R’_0_ and R’’_0_ are the nitrogen elimination rates (ml/minute) at time 0 in the 2 different curves, and k_1_ and k_2_ are time constants relating to nitrogen elimination rates. These were set at the averages derived from the clinical data in the original study: R’_0_ = 37.3, R’’_0_ = 13.9, k_1_ = 0.45, k_2_ = 0.06 [12].

The equations were solved using a 4th order Runge-Kutta analysis using Matlab (MathWork, MA, USA), producing washout curves as a function of washout time (*P_DS_*(*t*)). FRC and LCI were calculated from the generated washout curves and compared to results obtained running the model with no nitrogen backflow (*F*(*t*) = 0). LCI are quoted as the measured LCI, which incorporates the measured FRC rather than that set by the model, since this most closely mirrors the clinical situation.

### Modelling the Effects of Nitrogen Excretion in Adults

We used the model to explore the effect of increasing ventilation heterogeneity and increasing DS on measured LCI and measured FRC. Typical values for dead space in children and young adults with and without CF were derived from two studies, both showing an increase in DS even in subjects with otherwise mild CF [16], [17].

Using these data, we modelled two specific scenarios:

1. Healthy adult, with an FRC = 3L, R = 10L/min, DS = 0.3, and both X and Y equal to 0.5 (i.e. no ventilation heterogeneity).
2. CF adult with FRC = 3L and R = 10L/min as above, but with DS = 0.45, and both X and Y equal to 0.7.

In both of these cases we looked at the end of the washout, where the expired nitrogen concentration is 1.975% (1/40^th^ of the starting value) to assess the contribution to this signal made by excreted nitrogen. We also assessed the impact of nitrogen excretion on accuracy of FRC and LCI measurements, where the true FRC was taken as set in the model (3L) and the true LCI was calculated from a washout in which there was no nitrogen excretion.

### Modelling the Effects of Nitrogen Excretion in Children

Since nitrogen washouts are predominantly applied in paediatric populations, it is important to also explore the impact of nitrogen excretion in a child lung model. In order to model the effects in a 10yr old child, FRC and ventilation rate (R) were scaled down to 2 and 7L respectively. The data on nitrogen excretion, however, were derived from healthy resting adults, and will be affected by two different components in children that cannot be simply scaled on weight alone. The amount of nitrogen available for excretion within the first few minutes will be reduced in proportion to the lower mass of body tissue and blood volume in children [18]. On the other hand, the relative increase in cardiac output (in relation to total blood volume) means that tissues and lungs are better perfused, and this may serve to increase nitrogen excretion. The former effect would result in a reduction in nitrogen excretion by a factor of 0.45, whilst the latter would result in an increase in nitrogen excretion by a factor of 1.6 [18]. The true value is likely to lie between these two extremes, though in the absence of clinical data in children we have not attempted to extend the model further than defining these extremes.

The following two additional clinical scenarios were therefore modelled:

1. CF child, with conservative nitrogen excretion. FRC = 2L, R = 7L, DS = 0.5, X = 0.7, N_2_ excretion multiplied by 0.45.
2. CF child, with increased nitrogen excretion. FRC = 2L, R = 7L, DS = 0.5, X = 0.7, N_2_ excretion multiplied by 1.6.

In both cases we assessed the contribution of excreted nitrogen to total nitrogen concentration at end of washout and impact on measured FRC and LCI.

## Results

The effect of nitrogen excretion on nitrogen concentration at end of washout, LCI and FRC are summarised in Table 1 for a healthy adult model (no ventilation heterogeneity, DS = 0.3) and for an adult with both ventilation heterogeneity (X = 0.7) and increased DS (0.45). The two CF child scenarios are also summarised in Table 1.

**Table 1:** Effect of nitrogen excretion on LCI and FRC for different lung model scenarios.

	FRC (L)	R (L/min)	DS	X	LCI, LCI (N_2_)(% error)	FRC, FRC (N_2_)(% error)	Contribution of excreted N_2_ at LCI point (%)
**Healthy adult**	3	10	0.3	0.5	5.27, 5.50 (4.4)	3.00, 3.05 (1.8)	21%
**CF Adult**	3	10	0.45	0.7	8.96, 9.45 (5.5%)	3.00, 3.09 (3.1)	23%
CF child-Reduced N_2_*	2	7	0.5	0.7	9.64, 10.2 (5.8)	2.00, 2.06 (3.08)	24%
**CF child-Increased N_2_^+^**	2	7	0.5	0.7	9.64, 10.93 (13.4)	2.00, 2.14 (7.1)	49%

For the two CF child scenarios: * - N_2_ excretion reduced by a factor of 0.45; ^+^- N_2_ excretion increased by a factor of 1.6.

FRC: functional residual capacity, R: minute ventilation, DS: deadspace, X: ventilation heterogeneity (fractional ventilation distribution), LCI: lung clearance index.

### Impact of Nitrogen Excretion on Washout Progress

**Figure 2:**
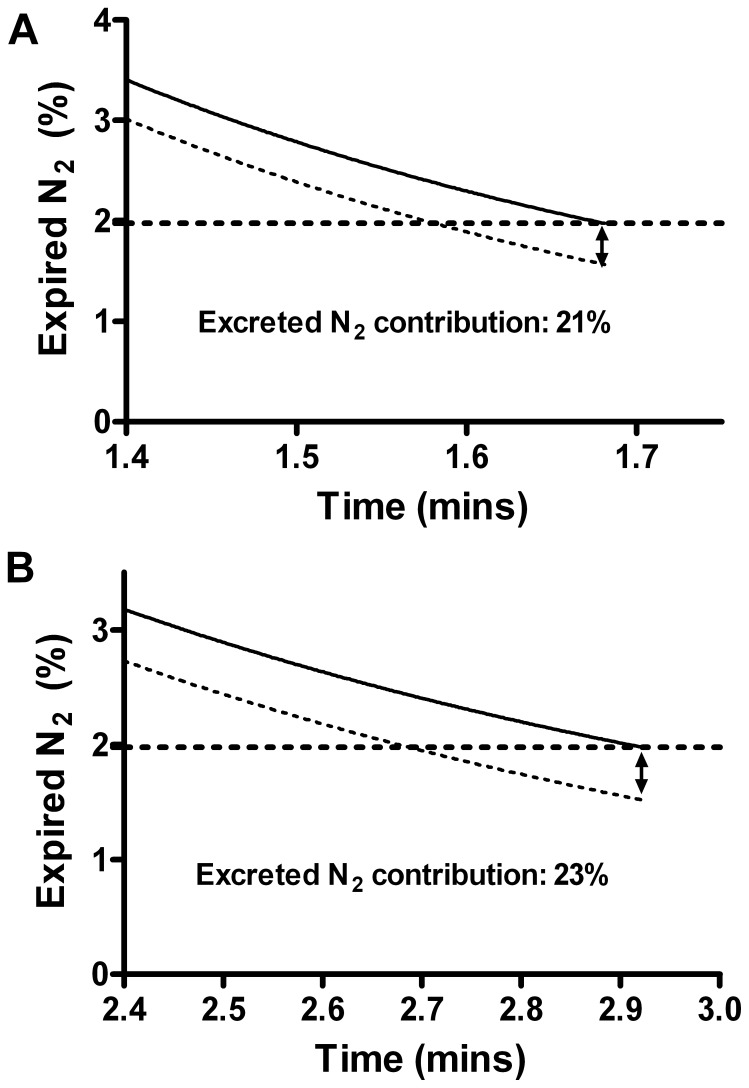
Effect of nitrogen (N_2_) excretion on expired N_2_ concentration from a two compartment lung model. Expired N_2_ is shown with (solid line) and without (dotted line) N_2_ excretion for a model representing a healthy adult (A), and a CF adult (B). Washout ends at expired N_2_ = 1.975% (1/40^th^ of the starting concentration). Increase in expired N_2_ at the true LCI point as a result of excreted nitrogen is indicated by the arrow.

The contributions of excreted nitrogen to the total measured nitrogen concentration at the end of a washout for the healthy and CF adult models are shown in Figure 2. At this point, when the total expired nitrogen signal is 1.975%, excreted nitrogen contributes 21% and 23% of the total nitrogen signal in the healthy and CF adult lung models respectively.

### Impact of Increasing Dead Space on Washout, and Effect of Nitrogen Excretion

**Figure 3:**
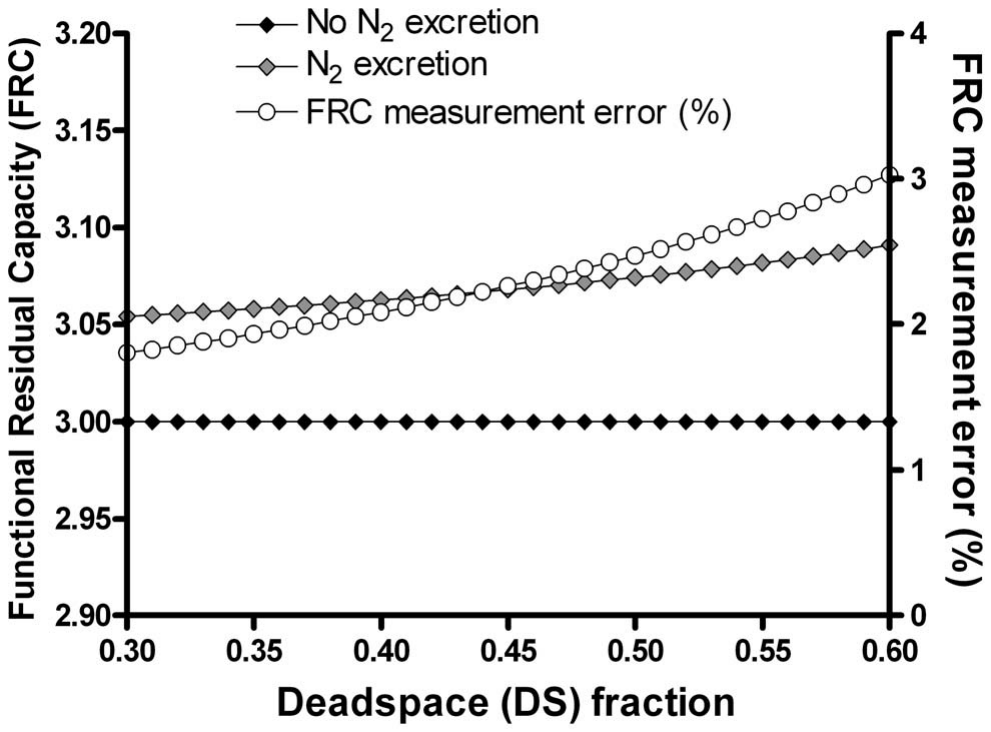
Impact of nitrogen excretion and increasing dead space fraction on functional residual capacity. In the absence of nitrogen (N_2_) excretion, increasing dead space did not change the measured FRC (black diamonds). The effect of N_2_ excretion on FRC and the percentage error in measured FRC are shown by the grey diamonds and open circles respectively.

For a typical adult subject, with an FRC of 3L (i.e. V_A1_+ V_A2_ = 3), the effect on measured FRC of increasing DS between 0.3 and 0.6 is shown in Figure 3 (and summarised in Table 1). There was a small absolute and percent effect of nitrogen excretion on measured FRC, with an error of 54 ml (1.8%) when DS was 0.3, increasing to 91 ml (3%) for DS 0.6.

**Figure 4:**
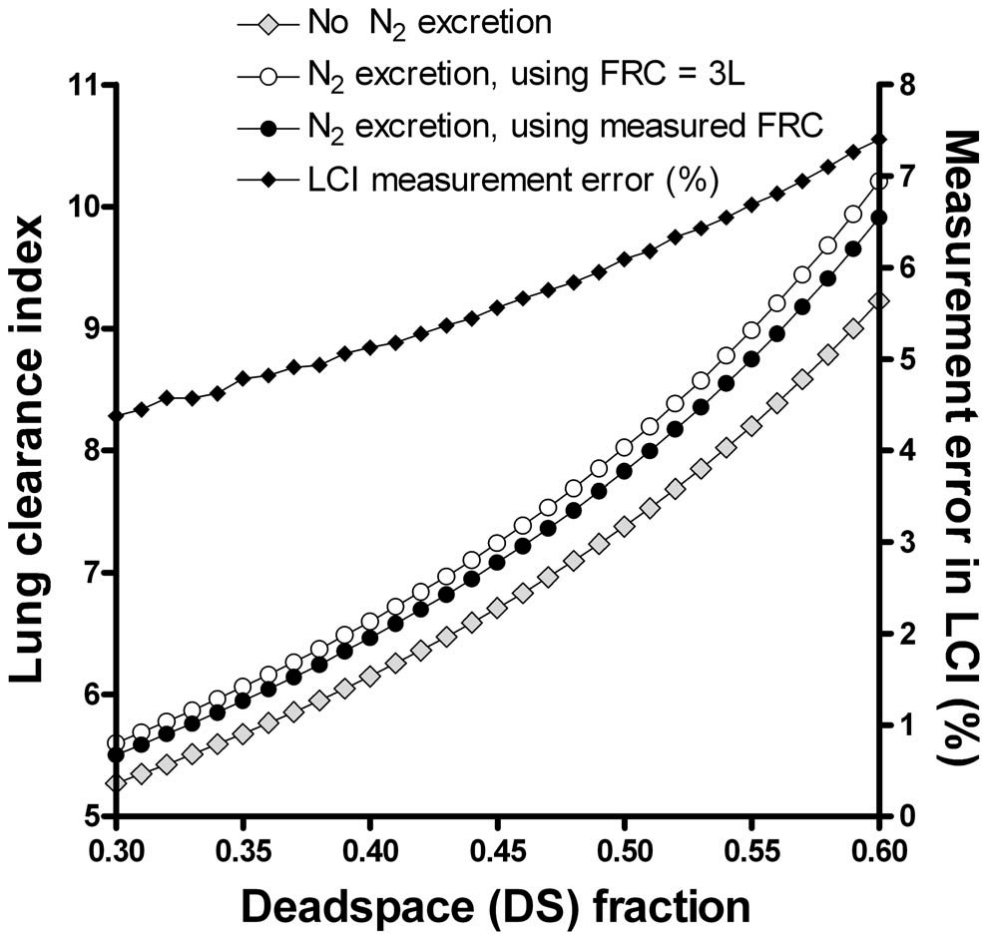
Impact of nitrogen excretion and increasing dead space fraction on lung clearance index. The impact of increasing deadspace on true LCI is shown by the lower curve (grey diamonds). The impact of nitrogen (N_2_) excretion on measured LCI is shown by the open circles, using true FRC. Measured LCI using measured FRC is also shown (black circles). The percent error in the measured LCI using measured FRC is shown by the black diamonds (right axis).

The impact on the LCI was partially offset by the error in FRC (since LCI is calculated from the cumulative expired volume at washout termination point, divided by the FRC), as shown in Figure 4. When this error was included, as it is in clinical measurement systems, then the LCI error was reduced from 6.3 to 4.4%. When DS was 0.6, excreted nitrogen caused an error in the LCI (after incorporation of the FRC error) of 7.4%, or 0.7 units.

### Impact of Increasing Ventilation Heterogeneity on Washout, and Effect of Nitrogen Excretion

**Figure 5:**
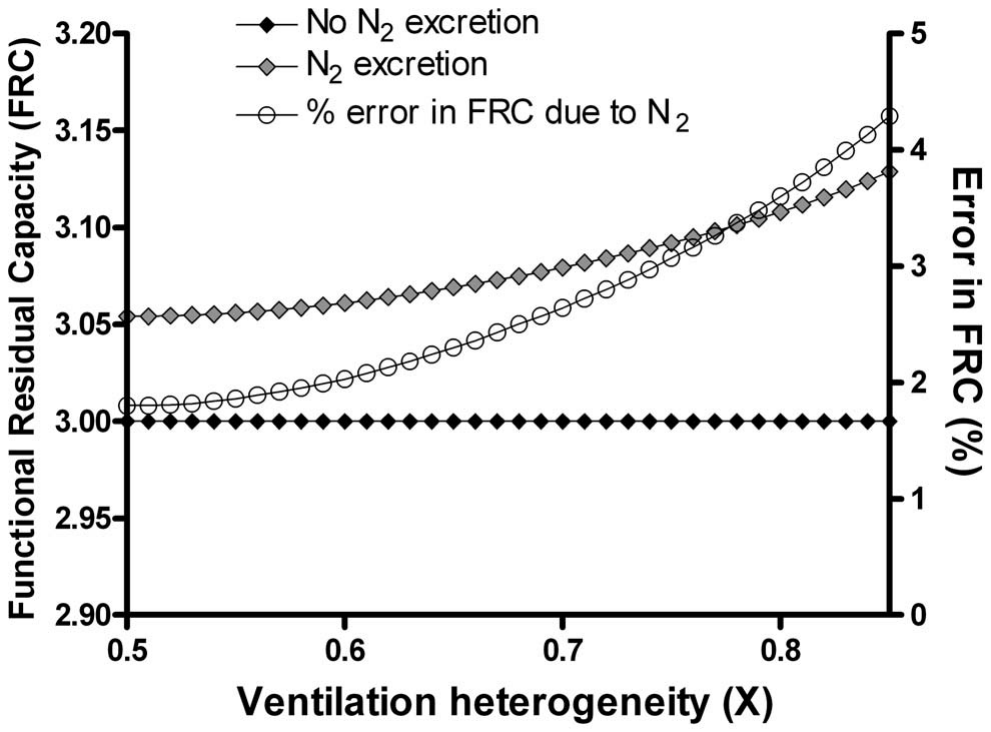
Impact of increasing nitrogen excretion and ventilation heterogeneity on functional residual capacity. In the absence of nitrogen (N_2_) excretion, increasing X did not change the measured FRC (black diamonds). The effect of N_2_ excretion on FRC and the percentage error in measured FRC are shown by the grey diamonds and open circles respectively.

**Figure 6:**
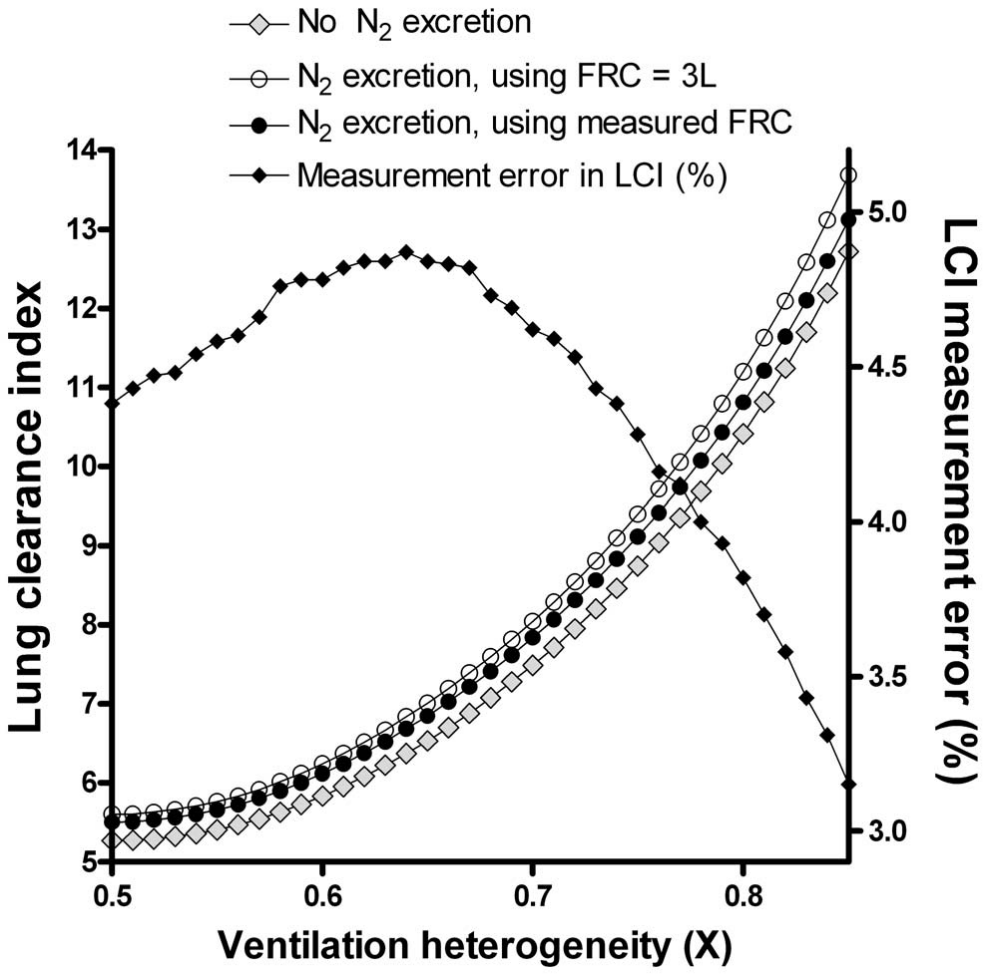
Impact of nitrogen excretion and increasing ventilation heterogeneity on lung clearance index. The impact of increasing X on LCI is shown by the lower curve (grey diamonds). The impact of nitrogen (N_2_) excretion on measured LCI is shown by the open circles. The error is partially offset for by the additional error in FRC (black circles). The percentage error in LCI caused by nitrogen excretion is shown by the black diamonds (right axis).

Separate to the effect of DS, increasing ventilation heterogeneity, here represented as X (the fractional distribution of ventilation), also had a major effect on LCI. Error in FRC increased from 50 ml (1.8%) when there was no ventilation heterogeneity to 129 ml (4.3%) when X was increased to 0.85 (i.e. lung compartment A2 received 85% of the ventilation), see Figure 5. The impact on the LCI was less predictable however, with an initial increase in LCI error to 4.9%, followed by a decline as ventilation heterogeneity continued to increase beyond X = 0.64 (Figure 6).

### Combined Effect of Increasing Dead Space and Ventilation Heterogeneity on Washout Parameters

**Figure 7:**
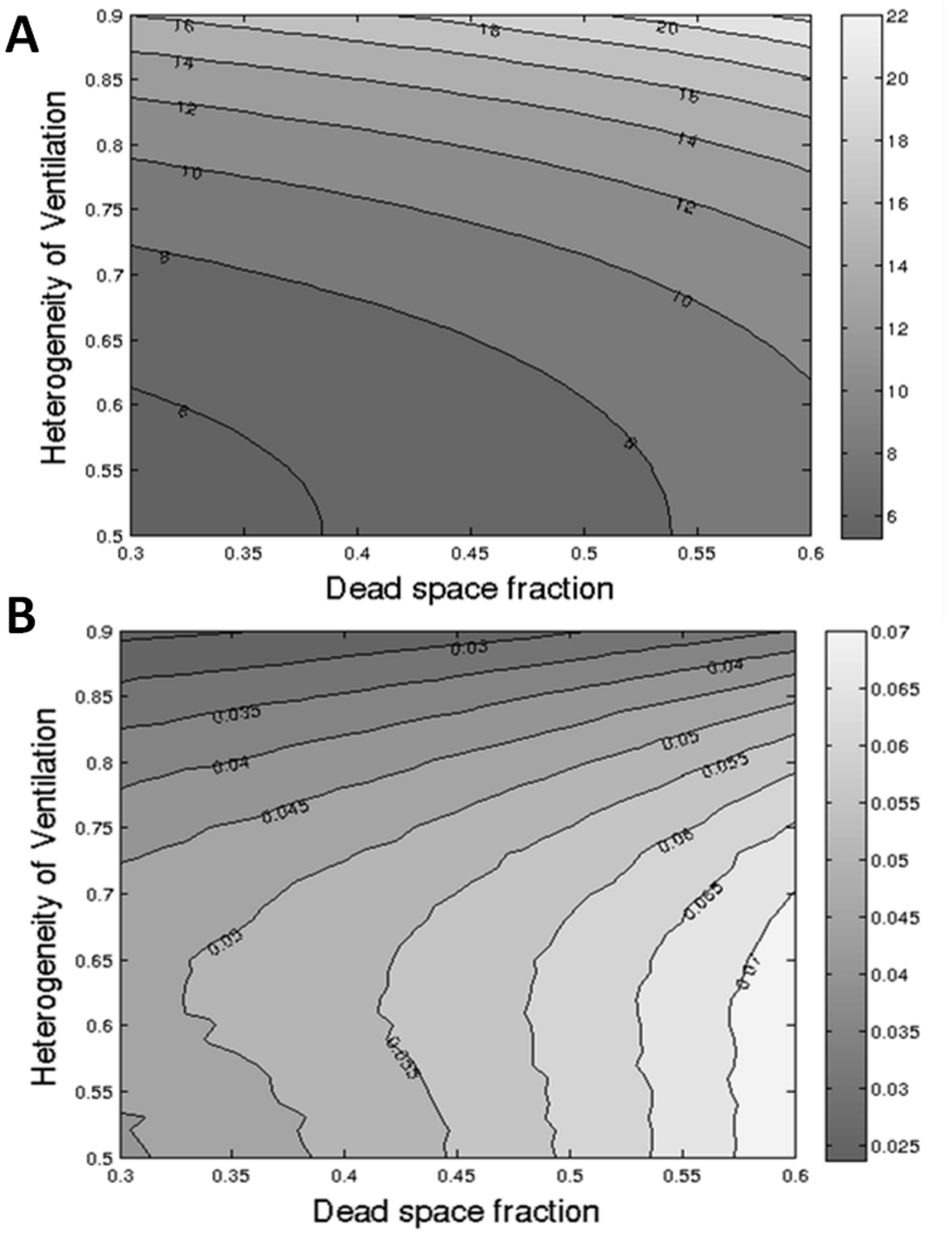
Contour map showing combined impact of nitrogen excretion, ventilation heterogeneity and dead space on nitrogen washout. Impact of increasing ventilation heterogeneity (varying X between 0.5–0.9) and dead space (0.3–0.6) is shown on LCI (A) and the fractional error in LCI caused by nitrogen excretion (B). Numbers on contours represent the resulting LCI (top) or fractional error (bottom). Fractional error of 0.07 (pale grey) represents a 7% error in LCI due to nitrogen excretion.

In real life, the effects of DS and ventilation heterogeneity are combined. For the adult lung model the combined effect of altering these two variables on LCI and error in LCI are shown in Figure 7.

### Impact of Nitrogen Excretion in Child Lung Model

**Figure 8:**
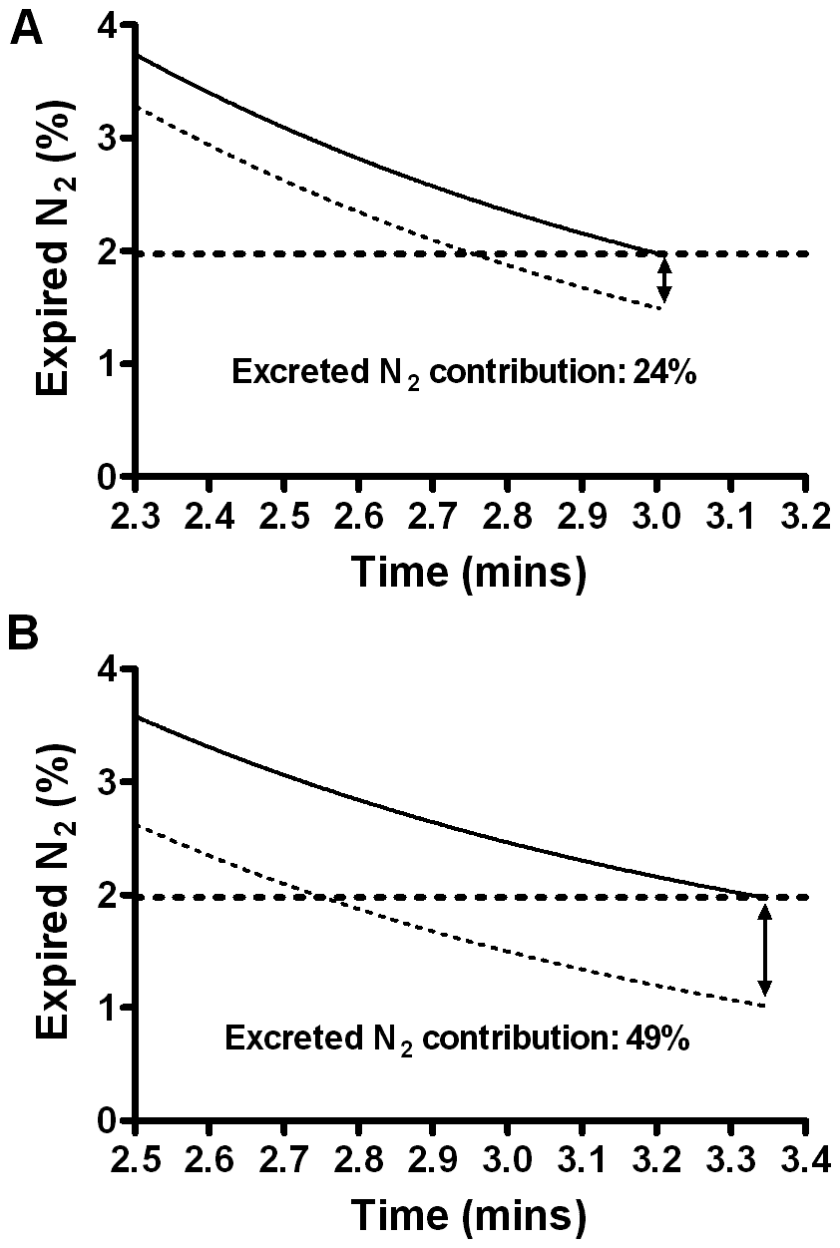
Contribution of excreted nitrogen to expired nitrogen concentration from a child lung model. Expired nitrogen (N_2_) is shown with (solid line) and without (dotted line) N_2_ excretion for a two compartment lung model adjusted to represent a child with cystic fibrosis. N_2_ excretion has been reduced by a factor of 0.45 (A) to reflect the lower body mass and blood volume, or increased by a factor 1.6 (B) to reflect increased tissue perfusion and relative cardiac output. Washout ends at expired N_2_ = 1.975% (1/40^th^ of the starting concentration). Increase in expired N_2_ at the true LCI point as a result of excreted nitrogen is indicated by the arrow.

The effects of two extremes of nitrogen excretion modelling are summarised for the CF child model in Table 1. The true errors in measured washout outcomes are likely to lie between the extremes described here: for LCI between 6 and 13%, and for FRC between 3 and 7%. The impact of these levels of nitrogen excretion on the washout signal are shown in Figure 8: excreted nitrogen contributes between 24 and 49% of the total final nitrogen concentration in this model.

## Discussion

With this lung model using existing experimental data on nitrogen washout kinetics, we have demonstrated that excreted nitrogen may make a substantial contribution to the measured nitrogen concentration during a multiple breath nitrogen washout. We have also shown that this contribution is variable, being increased by both of the major physiological processes known to affect LCI. The extent of this effect, causing a relative increase in nitrogen signal at the LCI point of over 20%, even in scenarios intended to reflect the processes in healthy lungs, is far beyond the +/−5% nitrogen concentration measurement error considered acceptable from a technical point of view [8]. Whilst the impact on FRC and LCI are less, varying dead space and ventilation heterogeneity in disease make the contribution of excreted nitrogen hard to calculate and predict. In scenarios designed to model the situation in a child with CF, the increase in LCI is between 6 and 13% as a consequence of excreted nitrogen. The true impact on LCI is even greater, but this is offset to an extent by the error in the FRC measurement, which serves to reduce the measured LCI.

This study represents the first attempt to understand the impact of excreted nitrogen on MBNW in disease. Kjellman described MBNW in healthy children, and also reported that any error due to excreted nitrogen was both small and less than that due to technical factors associated with the apparatus in use at that time [14]. His correction equations for clinical data, using the height of a typical 10 year old child (145 cm), would predict an error in FRC of 3.8% and in LCI (corrected for the erroneous FRC) of 0.8%. This small effect on FRC in healthy subjects is very similar to that which we have modelled, and supports the author’s conclusions that it is probably unnecessary to attempt to incorporate correction for nitrogen excretion in health. Crucially however, this only applies to those with healthy lungs, and the situation in disease in considerably more complex. Prolonged washouts in those with airway disease, with both increases in dead space and ventilation heterogeneity, mean that body nitrogen has longer to be washed from the lungs. In addition, we have very little understanding of how and where this nitrogen will be washed from. It may for instance be coming predominantly from the best ventilated regions, which rapidly achieve a high alveolar oxygen concentration and hence contribute the greatest nitrogen gradient. Our own modelling shows that the LCI error falls as ventilation heterogeneity alone increases, though the combined impact of increasing dead space and ventilation heterogeneity together is more complex.

Both the modelling here, and a recent clinical paper comparing multiple breath washout using nitrogen with that using exogenous SF_6_, support the presence of a significant but hard to predict effect of body nitrogen. In the study by Jensen *et al.*, there were no significant differences between FRC and LCI generated by nitrogen and SF_6_ washouts in healthy subjects [15]. In CF, as predicted here by the lung model, these differences were far more profound, and seemed to relate to underlying disease severity. The authors speculated that these differences might be related to the release of nitrogen from poorly ventilated lung regions that were not being adequately detected during the SF_6_ washin. The model used in this study is not able assess this explanation but the data support a critical and previously under-estimated role for body nitrogen. The reported difference in average LCI of 1.24 units (or 12%) in CF patients, is very similar to the 6–13% effect we have predicted for a similar (average) subject with CF.

In the model used in our study, air flow was modelled as a continuous flow, a simplification that both permits the use of the continuous equations described by Lundin [12], and also increases accuracy of the model. With a tidal system, end tidal expired nitrogen falls in a stepwise fashion, reducing the accuracy of estimation of the effect and potentially increasing LCI if the washout is extended by a whole step when less than this is required. Importantly the model generates measures of LCI similar to those seen in clinical practice [2] and volumes of excreted nitrogen that reflect both those described by Pendergast *et al.* [13] and predicted by Wanger *et al.* [19]. A two compartment model is clearly a simplification of what happens in the real lung, where there are multiple regions with different dead spaces and time constants. This is a necessary simplification however, in line with previous attempts to model gas mixing physiology [20]. Finally, we have assumed that ventilation and perfusion are matched, though we also observed that changing this had little impact on the overall conclusions (data not shown).

The experimental data we have used to generate the nitrogen excretion model are from adults, and we have been cautious in applying these to the very different physiology seen in children. The total amount of nitrogen stored in blood and tissue stores is less in smaller children. The increased cardiac output and tissue perfusion seen in children however mean that nitrogen washout kinetics are likely to be increased, particularly over the first part of the washout. Although the different factors in the washout equation could be individually adjusted, in our opinion the effect of this would be to introduce too many additional assumptions into the model. We have instead defined the limits between which nitrogen excretion error lies, and have shown that even with a conservative approach there is still a significant contribution from excreted nitrogen. Although the true value may be greater than this, the aim of this child lung model was primarily to demonstrate the presence of the effect. This model does not permit a detailed correction for excreted nitrogen, but it does highlight that this component cannot simply be ignored.

The impact of cardiac output in disease may also be important in adults. The experimental data on nitrogen washout kinetics were obtained from resting subjects. The first few minutes of washout are expected to depend primarily on lung perfusion (i.e. cardiac output), which may be significantly higher than the normal resting value in patients who are unwell or under stress. Under such conditions nitrogen excretion would be increased but the exact time course is unknown.

Ultimately, an important question is how important this is in real life. If the nitrogen washout methodology performs a similar task to that performed using SF_6_, then should it matter whether the nitrogen comes from the lungs or crosses the alveolar membrane from the blood? In some regards this is correct: if we have a working system that measures a signal it doesn’t necessarily matter to the average clinician exactly how that is generated. But LCI is a relatively new measurement in clinical practice, and most of our experience in the literature has been based on systems using exogenous tracer gases. The key intervention studies [5], [6], [21], [22], [23], the longitudinal studies [24] and those employing structural assessments alongside LCI [22], [25] all used SF_6_ as the tracer gas. These conclusions cannot be automatically applied to nitrogen washouts, regardless of how tempting this may appear. Our own data indicate that impact on LCI of excreted nitrogen is complex, and it cannot be assumed that reducing ventilation heterogeneity will automatically reduce the body nitrogen contribution (and hence increase the treatment effect) - as shown in Figure 7 the reverse may in fact be true. Added to this, there are the additional effects of 100% oxygen on gas mixing and ventilation-perfusion matching in the lung, which remain unexplored and ill-understood. All this should not detract from the use of LCI as a trial and clinical measurement, but these data sound an important warning note: considerable caution should be applied in applying conclusions derived from using one technique to another.

## Conclusions

In this lung model analysis, based on experimental nitrogen washout data, we found a significant impact of nitrogen excreted across the alveolar membrane on the measured nitrogen concentration. This effect was particularly strong during the last part of the concentration curve, where at least 20% of measured nitrogen originated from blood and tissues. Impact on accuracy of measured LCI and FRC was not constant and was affected by ventilation heterogeneity and dead space. Due to lack of paediatric nitrogen excretion data the effect in children is unclear and needs to be investigated further.
